# Soft Material-Enabled, Flexible Hybrid Electronics for Medicine, Healthcare, and Human-Machine Interfaces

**DOI:** 10.3390/ma11020187

**Published:** 2018-01-24

**Authors:** Robert Herbert, Jong-Hoon Kim, Yun Soung Kim, Hye Moon Lee, Woon-Hong Yeo

**Affiliations:** 1 George W. Woodruff School of Mechanical Engineering, College of Engineering, Georgia Institute of Technology, Atlanta, GA 30332, USA; rherbert7@gatech.edu (R.H.); ysk@me.gatech.edu (Y.S.K.); 2 School of Engineering and Computer Science, Washington State University, Vancouver, WA 98686, USA; jh.kim@wsu.edu; 3 Functional Materials Division, Korea Institute of Materials Science (KIMS), 797 Changwondaero, Seongsan-gu, Changwon, Gyeongnam 641-831, Korea; hyelee@kims.re.kr; 4 Center for Flexible Electronics, Institute for Electronics and Nanotechnology, Bioengineering Program, Petit Institute for Bioengineering and Biosciences, Neural Engineering Center, Georgia Institute of Technology, Atlanta, GA 30332, USA

**Keywords:** soft materials, flexible hybrid electronics, wearable electronics, stretchable electronics, medicine, healthcare, human-machine interfaces

## Abstract

Flexible hybrid electronics (FHE), designed in wearable and implantable configurations, have enormous applications in advanced healthcare, rapid disease diagnostics, and persistent human-machine interfaces. Soft, contoured geometries and time-dynamic deformation of the targeted tissues require high flexibility and stretchability of the integrated bioelectronics. Recent progress in developing and engineering soft materials has provided a unique opportunity to design various types of mechanically compliant and deformable systems. Here, we summarize the required properties of soft materials and their characteristics for configuring sensing and substrate components in wearable and implantable devices and systems. Details of functionality and sensitivity of the recently developed FHE are discussed with the application areas in medicine, healthcare, and machine interactions. This review concludes with a discussion on limitations of current materials, key requirements for next generation materials, and new application areas.

## 1. Introduction

Flexible hybrid electronics (FHE), configured in low-modulus, compliant materials via hard-soft materials integration, offer distinct advantages over the conventional electronic systems that are made of bulky and rigid materials. Recent advancements in the advanced materials and soft mechanics have enabled a successful integration of rigid, yet miniaturized chips with flexible/stretchable circuit interconnects, while maintaining low levels of effective moduli [1,2,3]. Thus, such FHE in wearable or implantable configurations can achieve a wide range of device functionalities via incorporation of capabilities of signal filter/amplification, analog-to-digital conversion, microcontroller, memory, and wireless power transfer in the systems [4,5,6]. In particular, FHE allow in vivo, continuous, and real-time monitoring of human health via conformal and tissue-friendly lamination on biological tissues, such as soft human skins [7,8,9] and internal organs with time-dynamic motions [10,11,12].

A successful development of high performance FHE requires thorough understanding of key materials, mechanics design, and advanced manufacturing processes. Specifically, engineering of material properties such as adhesion, flexibility, stretchability, and biocompatibility is critical for conformal and intimate integration with targeted human tissues. In general, FHE involve various types of sensors, circuits, and substrate materials; the combination of multiple components decides the overall mechanical and material properties of the electronics. Recent advances in soft materials, nano-microfabrication, and low-profile electronics have enabled the development of wearable and implantable electronics. The progress in materials processing and engineering of low-modulus functional materials allowed to design mechanically compliant electronics. Furthermore, hybrid integration of nanomaterials enhanced the electrical properties of the flexible and stretchable devices. As an example, carbon nanotubes (CNTs), first discovered in 1991 and synthesized in 1993, were embedded in soft elastomers for improved conductivity [13,14,15]. Graphene, a more recently observed material, is an emerging choice for creating conductive composites, while offering enhanced stretchability [16]. These materials, and others to be discussed in this review, offered versatility to manipulate a device’s electrical and mechanical properties, depending on target applications. In particular, the first generation of skin-like electronics, introduced in 2011 [17], opened a new era to develop unobtrusive and wearable sensors, actuators, antennas, and other components. These advances in materials and associated integration technologies have ignited the development of new FHE where high-performance electronic components are hybridized with soft materials.

Existing reviews of wearable and implantable electronics typically offer narrow scopes. For example, recent reviews [18,19] focus only on specific wearable devices such as strain or tactile sensors, while others [20,21] include expansion of wearable sensor types but still miss the coverage of important aspects of implantable FHE. Similarly, a few reviews summarize limited areas of implantable devices for neural applications [22,23], while emphasizing characteristics of particular materials, such as graphene, nanomaterials, hydrogels, and typical polymers [24,25,26,27,28]. Here, we provide a broader view of FHE via the comprehensive summary of the widely used functional materials to design soft wearable and implantable electronics, device integration strategies, and their applications in the areas of medicine, healthcare, and human-machine interfaces. In addition, we review the characteristics of those soft-material based electronics in terms of device type, sensing material, target signal, sensitivity and more. Overall, this review delivers a comprehensive summary of key materials, design criteria, and associated device performance to design high performance, wearable and implantable FHE.

## 2. Flexible Hybrid Electronics (FHE)

### 2.1. Definition

FHE refer to mechanically flexible and stretchable electronic devices and systems, enabled by hybrid combination of soft functional materials, compliant membranes, and integrated functional chip components. With the use of such materials, mechanical reliability with high flexibility and stretchability in the electronic devices can be achieved unlike traditional rigid electronics. As a result, FHE have found new applications in medicine, healthcare, and machine interfaces, via intimate and conformal interactions with soft and deformable human tissues. Recent advancement and current research in the development, characterization, and applications of a variety of FHE are discussed in the following sections.

### 2.2. Material Characteristics for Wearable and Implantable Electronics

Low-profile, wearable and implantable electronics can realize continuous, real-time monitoring of biomarkers for rapid, point-of-care disease diagnosis and therapeutics [29,30]. In terms of the device lamination on the human skin, poor contact leads to unreliable signal quality, contamination, and discomfort [7,9]. Thus, a careful selection of materials along with the mechanical analysis is critical to ensure the conformal and intimate contact of the device with the skin. Utilization of soft materials offers enhancement of the device’s overall flexibility. A systematic engineering of a thickness, stiffness, and modulus of materials provides proper mechanical compliance to make the device lamination on contoured and deformable geometries with enough adhesion. Mechanical flexibility and stretchability is generally achieved by either a deterministic structure design or hybrid material’s composition [31,32]. In a deterministic system, serpentine and wavy-structured interconnects of rigid materials can accommodate applied strain, while an island-bridge geometry utilizes these interconnects to isolate strain on the rigid-material embedded island. A randomly oriented composite integrates functional materials with intrinsically soft organic materials such elastomers [33]. Deterministic systems are advantageous in the design of high-performance electronics, while composite material-enabled systems offer higher mechanical compliance. In addition, electrical properties such as conductivity, linearity, sensitivity, and selectivity are important factors for achieving high-quality and reliable sensor performance. High sensitivity of wearable and implantable devices is necessary to detect subtle changes of target analytes or physiological signals. Sensitivity relies on the electrical properties, material’s composition, and structural design of the sensing materials. Similar to mechanical integrity, minimizing hysteresis is also required to prevent changes in electrical properties or responses under mechanical deformation on the living body. Another important factor to consider for flexible bioelectronics is biocompatibility, hemocompatibility, and biodegradability of materials. Adverse reactions between electronic materials and human tissues should be avoided for safe and long-term use. In particular, bioresorbable capability of transient materials can make a next-generation implantable device that avoids follow-up surgeries.

### 2.3. Sensing Materials

In wearable and implantable sensors, flexibility and stretchability are often limited by the sensing material, as these materials are significantly more rigid than substrate materials. As a result, informed selection of materials and structures for the sensing component are necessary to improve mechanical and electrical characteristics. Table 1 contains a summary of common and emerging sensing or active structures and materials for FHE.

**Figure 1:**
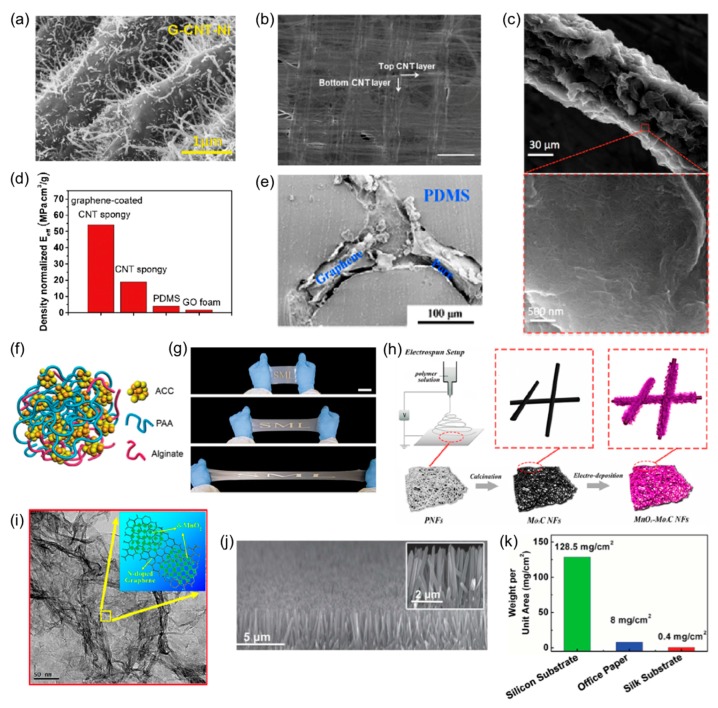
Soft functional materials. (**a**) Graphene-CNT-nickel hetero-nanostructure. Reprinted with permission from Reference [34], Copyright 2017, John Wiley and Sons; (**b**) Cross-stacked graphene-CNT (carbon nanotube) films. Reprinted from Reference [2], Copyright (2017), with permission from Elsevier; (**c**) SWCNT-based nanowire. Reproduced from Reference [35] with permission of The Royal Society of Chemistry; (**d**) Graphene oxide foam with low-effective elastic modulus for high sensitivity. Reprinted from Reference [36], Copyright (2017), with permission from Elsevier; (**e**) Graphene network embedded in PDMS. Reprinted with permission from Reference [37], Copyright 2016 American Chemical Society; (**f**) Schematic of mineral hydrogel. Reprinted with permission from Reference [38], Copyright 2017, John Wiley and Sons; (**g**) Elongation of 0%, 250%, and 500% of dielectric, liquid metal embedded elastomer. Reprinted with permission from Reference [39], Copyright 2016, John Wiley and Sons; (**h**) Process of developing MnO_2_-Mo_2_C nanofiber film. Reprinted with permission from Reference [40], Copyright 2016 American Chemical Society; (**i**) Nitrogen-doped graphene-MnO_2_ nanosheet composite. Reprinted with permission from Reference [41], Copyright 2016 American Chemical Society; (**j**) Cross-sectional view of aligned NW array. Reprinted with permission from Reference [42], Copyright 2016, John Wiley and Sons; (**k**) Chart indicating silk fibroin as an ultra-lightweight substrate. Reprinted with permission from Reference [43], Copyright 2016, John Wiley and Sons.

As one well-known sensing material, CNTs are cylindrical tubes that are categorized as single-walled (SWCNTs) and multi-walled (MWCNTs). Due to high conductivity and flexibility, CNTs are used in a variety of structures and composites to improve mechanical or electrical properties. Soft elastomers with poor conductivity can be altered to highly conductive composites with the addition of randomly oriented CNTs. The functional elastomers can act as conductive substrates to improve signal reception or other functions to build a soft actuator; an example is shown in Figure 1a [34]. CNTs can also be aligned in films to improve functionality in the desired direction [2]. When subjected to external strain, randomly oriented CNT structures first align in the direction of external stimulation, resulting in minimal initial resistance changes. An example of aligned CNT films is shown in Figure 1b. Furthermore, CNTs can be doped for specific applications, such as an ionic liquid doping of SWCNTs to make it more sensitive to light [63]. The SWCNT-based microwire in Figure 1c undergoes an electrical resistance change when strain, pressure, or torsion is applied [35]. Although this microwire is fragile, it is capable of rejoining to produce and recover electrical property changes. In addition to wires, CNTs are used to develop thin films. Such films, particularly when formed wrinkled on a soft substrate via heating of the film or a prestrained substrate, are highly stretchable and flexible. A single layer of thin film or a layer-by-layer assembly can be used [64]. Additionally, CNTs can be modified in multiple ways to achieve biocompatibility and biodegradability [44].

Another emerging active material is graphene. Graphene can be applied in a variety of forms, including porous foams, flakes, and thin films, while showing favorable electrical and mechanical properties. In general, graphene-based wearable electronics are less stretchable and flexible than CNT-based electronics. However, graphene is more biocompatible [47,65]. Figure 1d shows a graphene oxide (GO) foam developed with a low modulus for high sensitivity [36]. A number of such foams are embedded in soft substrates to develop piezoresistive elastomers (Figure 1e) [37]. Doping with GO improves electrical conductivity and mechanically stiffen structures [63]. Graphite flakes can act as a coating layer to improve properties as well [66]. Micro-fluid, based on GO sheets, is also used as sensing materials [67]. Printable, conductive designs have been configured with graphene nanoflake inks [68]. Hydrogels can be used as biodegradable sensing materials or substrate capable of high strain deformations and self-healing. As shown in Figure 1f, a hydrogel consists of amorphous calcium carbonate (ACC) nanoparticles, polyacrylic acid (PAA), and alginate chain crosslinks [38]. Another composite sensing material consists of a polyvinyl alcohol (PVA) and borax hydrogel containing SWCNTs, graphene, and AgNW [69]. Hydrogels can also provide ionic connections between electrodes [70].

Highly flexible and stretchable properties of the sensing materials may also be achieved with liquid metals. The most commonly used liquid metal is the inorganic and biocompatible eutectic gallium indium (EGaIn), which can maintain electrical conductivity under high strain conditions. EGaIn is often selected due to its favorable properties, including low toxicity and high conductivity [71]. As a sensing material, liquid metals are enclosed in microtubules or microchannels within soft substrates. Liquid metals can be dispersed in soft substrates to create conductive or dielectric elastomers (Figure 1g). EGaIn mixed with a low-modulus elastomer (Ecoflex) produces a high dielectric constant [39]. Magnesium oxide (MnO_2_) is a common material choice for nanosheets in supercapacitor applications. The process of creating an MnO_2_ nanosheet is shown in Figure 1h, and the structure of a nitrogen-doped MnO_2_ nanosheet is in Figure 1i. Mg films offer biodegradable electronic devices. Thin metal films, such as Au or Cu, can also be applied as electrodes using fractal, serpentine designs for improved flexibility and stretchability [72]. NWs are often adopted as sensing materials due to unique electrical and mechanical properties. Silver nanowires (AgNWs) patterning and films are demonstrated [63,73]. As shown in Figure 1j, aligned NWs can be grown on the substrate similar to CNTs. Carbon sponge has been incorporated into a PDMS substrate [74]. Conducting polymers, such as biocompatible PEDOT:PSS, allow textile-based wearable devices [75].

### 2.4. Substrate Materials

Unlike sensing materials, highly stretchable and flexible materials are generally used as substrates where sensing materials are embedded. Organic materials, compared to rigid inorganic ones, offer high flexibility, stretchability, and good adhesion for conformal lamination onto human tissues.

Table 2 summarizes a list of soft substrate materials and their mechanical properties, used for the development of wearable and implantable FHE. Silicone elastomers including Ecoflex, Sylgard, Dragon Skin, and Silbione are biocompatible in general and they are highly compliant with maximum elongation up to 900%. Strong adhesion of such elastomers onto target surfaces can be achieved in engineering of thin film formation. As noted in the previous section, elastomers may be combined with active sensing materials to improve electrical functionality while maintaining favorable mechanical properties. In addition to silicone elastomers, a variety of flexible polymers have been used, such as parylene, PET, PI, and poly(lactic-co-glycolic acid) (PLGA). In particular, PLGA is a common choice for biodegradable device applications [10]. These materials can also be applied as interlayers for better integration of specific materials with a substrate [76]. Additional substrates include paper, sellotape, and silk fibroin (biodegradable protein fiber). Among those, silk fibroin is used in ultra-lightweight and biodegradable applications, as indicated in Figure 1k [43].

### 2.5. Wearable FHE

**Figure 2:**
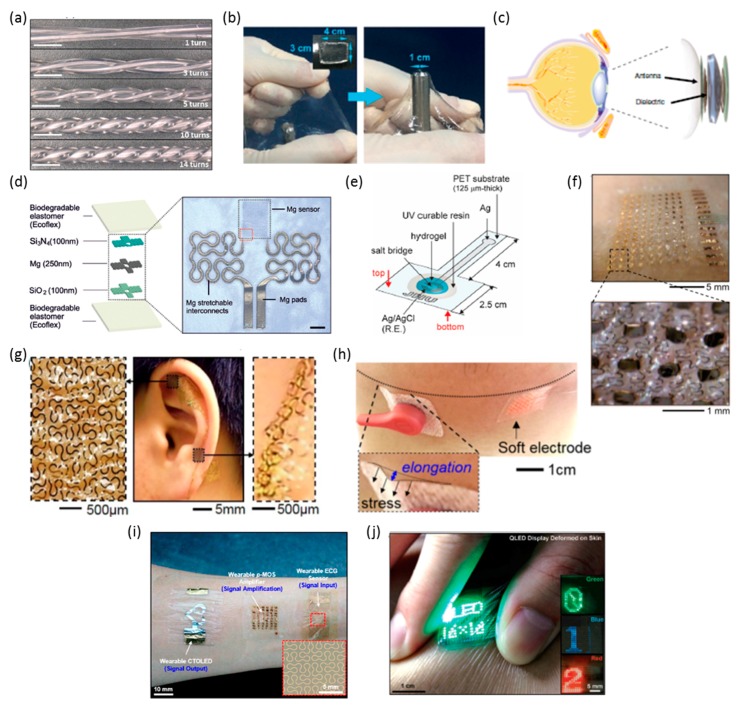
Wearable electronic systems. (**a**) Microtubule strain sensor and interconnect. Reprinted with permission from Reference [107]. Copyright 2017, Scientific Reports; (**b**) Self-healing strain sensor. Reprinted with permission from Reference [108]. Copyright 2017 American Chemical Society; (**c**) Wireless glucose and intraocular sensor. Reprinted with permission from Reference [29]. Copyright 2017, Nature Communications; (**d**) Biodegradable temperature sensor. Reprinted with permission from Reference [78], Copyright 2017, John Wiley and Sons; (**e**) Schematic illustration of a sweat chloride sensor. Reprinted from Reference [70], Copyright (2017), with permission from Elsevier; (**f**) EOG (electrooculography) electrode mounted on the skin. Reprinted from Reference [8], Copyright (2017), with permission from Elsevier; (**g**) EEG electrodes and interconnects on the auricle area [72]; (**h**) Comparison of rigid electrode with associated stress and soft material-enabled skin-like electrode. Reprinted with permission from Reference [109]. Copyright 2017, Scientific Reports; (**i**) Skin electrodes with amplifier. Reprinted with permission from Reference [110]. Copyright 2017 American Chemical Society; (**j**) QLED (quantum dot light-emitting diode) display on the human skin. Reprinted with permission from Reference [111], Copyright 2017, John Wiley and Sons.

Soft material-enabled, wearable electronic devices and systems include skin-mounted non-invasive sensors, soft actuators, flexible displays, low-profile energy storage systems, and miniaturized wireless communication devices. Figure 2 highlights a variety of wearable FHE that were used for conformal integration with human skins for monitoring of mechanical strain, pressure, and biopotentials and displaying information on contoured geometries. Table 3 reviews and summarizes device type, sensing materials, substrate materials, applications, target signals, and their mechanical and functional characteristics for wearable applications.

#### 2.5.1. Strain Sensors

Numerous strain sensors in wearable configurations have been developed, as highlighted in Table 3. Soft substrates and functional materials offer high stretchability and sensitivity of the designed strain sensors. They typically function by undergoing a change in electrical resistance when experiencing strain change. As a result, those sensors rely on the sensing material’s inherent characteristics. To design a highly sensitive resistance sensor, one study utilized SWCNT wires via wet spinning on a polydimethylsiloxane (PDMS) membrane [35]. This sensor also functioned as a pressure and torsion sensor. Although these microwires were fragile, the randomly oriented SWCNT networks fractured and rejoined under deformation, resulting in measurable electrical resistance changes with a high gauge factor (GF) of 10^5^ at 15% strain. The soft PDMS substrate aided in recovering the initial electrical resistance.

Another study achieved a very high GF by using graphene in a GO woven fabric (GWF) design based on a GO-coated cotton bandage template [117]. This strain sensor, encapsulated by a natural rubber latex, indicated a maximum elongation of 57% and a GF of 416 and 3667 at lower and higher strains, respectively. One work coated silk fibers with graphite flakes to develop a fiber-shaped strain sensor [66]. With a silk fiber, the sensor showed a GF of 14.5 up to 15% strain while a spandex fiber showed a GF of 14.0 up to 30% strain. As discussed previously, graphene can be manipulated in a variety of forms; one study used it as a force sensing material and contact electrode with PMMA coating [118]. This design achieved a GF of 42.2 for up to 20% elongation with ability to sense tension, bending, and torsion. Higher GFs, between 47.74 and 98.66, and a maximum elongation of 30% were reached with a graphene foam and PDMS composite [37].

To improve stretchability and maintain a high sensitivity, silver-coated polystyrene spheres (PS@Ag) were mixed with liquid PDMS to form printable electrodes and strain sensors [119]. With a 60 wt.% of PS@Ag, the sensor achieved a maximum elongation of about 80% and GFs, from lower to higher strains, of 17.5, 6, and 78.6. Whereas the previous CNT structure resulted in limited stretchability, the LBL assembly of SWCNT and thin polymer layers on a polycaprolactone (PCL) membrane created a piezoresistive, biocompatible strain sensor with a maximum strain of 100% and adjustable GFs between 5–13 [64]. While these previous strain sensors aim for high GFs, they sacrifice stretchability. To improve maximum elongation, a carbon sponge-PDMS composite was developed as a strain sensor to monitor pulse, breathing, and walking [74]. This composite sensor endured up to 200% strain and repeatable bending of 180°, and indicated a GF of 1.78 in tension. CNTs can also be designed to realize highly stretchable strain sensors. MWCNTs mixed with Ecoflex were printed into a partially cured substrate to produce a strain sensor capable of stretching 300% with a linear electrical response and GF of 1 [112]. Similarly, an Ecoflex and MWCNT nanocomposite configured a multidirectional strain and pressure mapping sensor with measurements based on anisotropic electrical impedance tomography [120]. This work applied the device as a human-machine interface, and possesses potential to develop large, irregularly shaped sensors.

In addition to embedding sensing materials into soft substrates, stretchable strain sensors can be developed by manipulating the material’s structure. By heating a CNT membrane, a wrinkled thin film was produced [121]. This wrinkled structure, integrated on an Ecoflex substrate, allowed conductivity up to 750% elongation, an approximate 60 times increase versus non-wrinkled films. GFs were 0.65 below 400% strain and increased to 48 above 400% strain, due to film fracture. Another material applied in stretchable strain sensors is liquid metal (EGaIn). One method of integrating EGaIn is to fill microtubules formed by soft polymers with the liquid metal [107]. These microtubules shown in Figure 2a can be used for contact measurements via resistance changes without electric failure upto 750% elongation. The use of other polymers, such as a PDMS nanocomposite, may improve the stretchability. Similarly, another study presents soft polymer microtubules filled with EGaIn to measure strain, torsion, and contact via capacitive changes [122].

Combining a self-healing hydrogel with SWCNTs, graphene, and AgNW realized a more stretchable piezoresistive sensor capable of enduring up to 1000% strain [69]. The hydrogel used here consisted of polyvinyl alcohol (PVA) and borax. This configuration achieved a GF of 0.24 at strains below 100% and of 1.51 at 1000% strain. Another strain sensor based on hydrogel used a PVA/polyvinylpyrrolidone (PVP) hydrogel with Fe^3+^ cross-linked cellulose nanocrystals (CNCs) to produce a strain sensor with 830% maximum elongation and self-healing ability [108]. The stretchability of this hydrogel is observed in Figure 2b. The CNCs-Fe^3+^ bonds act to dissipate energy when stretched and to improve toughness of the hydrogel. This strain sensor measured breathing and blood pulse. Wearable biaxial strain sensors have also been developed. As mentioned in the CNT discussion, aligned CNT arrays offer advantages over random orientation. With this understanding, one study developed a biaxial strain sensor with a GF of 3 via cross-stacked, aligned CNT films [2]. These CNT films were hybridized, or welded, with graphene to improve the biaxial sensing ability and increase the GF by at least 5 times existing cross-stacked CNT sensors. Similarly, aligned CNTs in polymer substrates improved functionality over randomly oriented composites [123]. This method produced a poly(methyl methacrylate) (PMMA) and PDMS composites for a multifunctional flexion- and tensile-sensitive electronic skin.

#### 2.5.2. Pressure Sensors

Pressure sensors can use capacitive or resistive-based measurement, depending on materials and design selections. Similar to strain sensors, graphene can be applied in a variety of forms. A capacitive pressure sensor used a GO-based low elastic modulus foam as the dielectric material to achieve a high sensitivity of 0.8/kPa [36]. Another capacitive pressure sensor used a dielectric layer of polyethylene (PE) film placed between two hydrogel films formed a capacitive pressure sensor and resulted in a sensitivity of 0.17/kPa [38]. This hydrogel can self-heal when two parts of hydrogel contact and is able to fully recover if dehydrated. One study configured a single layer piezo-capacitive pressure sensor with an AgNW pattern on polyurethane-urea (PUU) [124]. This sensor showed stability under 35% elongation and 10,000 cycles at 30% elongation. The Bao group developed a highly sensitive pressure sensor using flexible transistors [125,126]. Here, a microstructured PDMS acted as a dielectric layer, where the micropatterns were varied to tune pressure sensitivity as high as 8.4/kPa. Resistive pressure sensors have also been studied, and often function as strain sensors as well. A graphene porous network embedded in PDMS resulted in a pressure and strain sensor with sensitivities of 0.09/kPa and GFs of above 2.6, respectively [37]. Additionally, this sensor determined the frequency and degree of bending. Graphene-based micro-fluids can also be sensing materials, such as that formed by dispersing GO nanosheets in distilled water [67]. The microfluidic channel placed in an Ecoflex layer and PDMS substrate acted as a resistive strain and force sensor, with a sensitivity of 3.38^−2^/kPa. Similarly, other liquid conductive metals have been developed, such as that used in a resistive pressure sensor based on a microfluidic diaphragm [51,71]. This diaphragm constructs a Wheatstone bridge circuit, enabling radial and tangential strain detection based on the microchannel resistance changes with a sensitivity of 0.0834/kPa. One study indicated a method of remarkably increasing low pressure sensitivity of a pressure sensor from 02.57/kPa to 20.6/kPa by using GO to dope an electrospun polyurethane (PU) nanofiber membrane with PEDOT and PDMS encapsulation [63]. Graphene also can improve NW functionality when integrated with soft contact lenses (Figure 2c) to measure glucose and intraocular pressure with 25% stretchability and 91% transparency [29]. By adding graphene to the structure, resistance reduced during mechanical bending. The field effect transistor (FET) to sense glucose utilized a graphene channel as well. Another study integrated graphene with a contact lens for eye protection from electromagnetic waves and dehydration [127].

#### 2.5.3. Other Types of Sensors

In addition to strain and pressure sensors, many other types of wearable sensors exist for measuring temperature, light, sweat, and vibration. A biodegradable temperature sensor was composed of a thin Mg film active layer and Si_3_N_4_ and SiO_2_ dielectric layers, encapsulated by Ecoflex [78]. This biodegradable sensor in Figure 2d indicated a resistive sensitivity of 0.2%/K, stretched up to 10%, and bent to a radius of 1.75 mm. Multifunctional sensors also exist for wearable applications, as one study showed for measurement of temperature, pressure, and light with ionic liquid and Au film as sensing materials [63]. By doping the ionic liquid with SWCNTs, the ionic liquid experienced an increase in light sensitivity due to the CNTs conversion of light energy to heat. By applying a InGaZnO thin film in an ion-sensitive field-effect transistor (ISFET), one study designed a sensor for simultaneous sweat pH and skin temperature monitoring to withstand bending to a 1 cm radius [114]. This ISFET on a PI substrate acted as a pH sensor while a CNT-PEDOT:PSS solution acted as the temperature sensor, both of which were incorporated onto a PET substrate. Another sweat sensor, shown in Figure 2e, measured sweat chloride and incorporated hydrogel [70]. The agarose gel acted as a salt bridge between two electrodes, creating an ionic connection between a reference solution and the environment while preventing equilibration. The Wang group applied a temporary tattoo-based sweat sensor to measure lactate [128]. Additionally, they integrated a lactate sensor with electrocardiogram (ECG) electrodes into a single patch and confirmed minimal interference [129]. In a similar way, they manufactured a tattoo-like glucose sensor and achieved a sensitivity of 23 nA/µM [130]. This group also investigated stretchable electrochemical sensors for other wearable applications [131]; PEDOT:PSS and Ag/AgCl inks, printed on an Ecoflex substrate exhibited up to 100% linear and 150% radial stretching. Additionally, a work developed a colorimetric humidity sensor via a nanowire cluster film (NWCF) [132]. Fabricating this cluster film required sputtering on a disordered anodic alumina oxide substrate, rather than a normal flat substrate, and the resulting NWCF bent to a 1.85 cm radius and indicated stability over stretching and bending cycles. The result could be visualized as NWCF exhibited an extinction spectrum that varied with water, or sweat, content on its surface. Lastly, a vibration sensor used a cellular structured graphene composite elastomer with highly soft and piezoresistive properties. This sensor detected frequencies between 300–20,000 Hz and worked as an accelerometer with a linear electrical response [133].

#### 2.5.4. Electrodes

Another wearable device are non-invasive, skin-mounted electrodes for recording biopotentials for human health monitoring, disease diagnostics, and machine interfaces. One device used a triple-layer graphene electrode array with a graphene monolayer ground [134]. This electrode array allowed 8% and 15% elongation in separate directions, and detected approaching objects up to 7 cm away due to absorption of the object’s electric field. Another study developed flexible, biocompatible microelectrodes by patterning AgNWs onto a hydrogel substrate of PEG, agarose, and PAAM, allowing flexibility to bend up to a 10 mm radius [73]. By patterning thin Au on PI substrates, electrooculography (EOG) electrodes can be developed to withstand over 30% strain and 500 micron bending radius [8]. This skin-like electrode, shown in Figure 2f, aimed to allow controlling a wireless wheelchair via EOG signals. Similarly, a study implemented a fractal design of Au for conformal contact of an electroencephalogram (EEG) electrode to the skin (auricle area; Figure 2g), which was used for a persistent brain-computer interface (EEG-based text speller) [72]. Figure 2h provides a comparison of a rigid-material based electrode and a soft material-enabled, stretchable electrode for electromyography (EMG) recording on the chin [109]. Unlike the stress-inducing rigid electrode, the skin-like sensor showed comfortable, intimate contact onto the skin. This electrode, built of Au nanomembrane on a silicone-PVA substrate, endured up to 150% strain and conformed to a 500 micron bending radius. A wearable textile-based keyboard, capable of withstanding 30% elongation, used a knitted textile substrate [75]. Areas of the substrate were coated with PEDOT:PSS to produce a capacitive electrode when touched with a human finger, allowing for application as a keyboard. CNTs can be applied to improve electrode functionality and performance. Embedding CNT networks into polymers created a wearable p-MOS inverter consisting of four CNT transistors for amplification of ECG signals from a wearable electrode and is shown in Figure 2i [110]. Another application of CNT is to make a CNT-PDMS composite for enhanced interface between the skin and ECG sensor [113]. This composite maintained flexibility of pure PDMS but incorporated ethoxylated-polyethylenimine (PEIE) to improve adhesion while the CNTs improved conductivity.

#### 2.5.5. Electrical Components, Displays, and Actuators

Beyond sensors and energy storage devices, soft materials enable wearable actuators and flexible passive electrical components. In particular, transmission lines in electronics have been developed. A conductive graphene ink consisting of graphene nanoflakes can be printed and compressed on a paper substrate as a transmission line [68]. This transmission line did not experience a change in transmission coefficients despite bending and twisting. Flexible antennas were similarly developed. Another method of creating a flexible and stretchable transmission line is via a wrinkled CNT film, formed by transferring CNT sheets to a prestrained elastomer [135]. After releasing the strained elastomer, the CNT layer produces a wavy shape. From this method, a stretchable conductive transmission line experienced minimal strain induced changes of electrical resistance up to 600%. Alumina passivation of the CNT sheets improved performance. One group developed a highly conductive printable elastomer of mixed PDMS and Ag powder for wearable wireless applications, including fabrication of a transmission line and RF antenna [115]. EGaIn can also form flexible and passive electrical components, such as resistors, inductors, and capacitors via a patterning process based on soft lithography [136]. With this structure, a 2.5 D circuit integration enabled to power two LEDs with 35% strain. Liquid metals can be embedded in soft elastomers to alter electrical properties, such as mixing EGaIn with Ecoflex to produce the stretchable dielectric elastomer in Figure 1g [39]. Silk fibroin film enabled an ultra-lightweight and biocompatible resistive switching memory device [43]. This device conformed to an 800-micron radius while maintaining functionality with a retention time above 10,000 seconds and on/off ratio of 100,000. A stretchable transistor from the Bao group [33,137] utilized a styrene-ethylene-butadiene-styrene hydrogenated elastomer as a dielectric and substrate material, along with electrodes and semiconductors that were composed of SWCNTs. This device, unlike other SWCNT transistors, showed great reproducibility. By testing different diameters of SWCNTs, the transistor indicated a maximum mobility of 15.4 cm^2^/Vs and an on/off ratio above 1000.

Wearable displays have also been developed with the usage of soft materials, such as a quantum dot light-emitting diode (QLED) display of ultrathin layers, including a layer of PEDOT:PSS encapsulated by parylene C and epoxy [111]. This display, shown in Figure 2j, bent around a radius of 68 microns and endured 1000 bending cycles. Furthermore, the study integrated the display into a touch interface, temperature sensor display, and a step counter. Another wearable display incorporated polymer light-emitting diodes (PLEDs) and organic photodetectors (OPDs) [138]. Again, ultrathin layers were encapsulated by a parylene substrate and the device conformed to a radius below 100 microns, stretch to 60%, and compress 67% while maintaining functionality. Wearable actuators have also been explored, including an artificial muscle consisting of the graphene-CNT-nickel (G-CNT-Ni) hetero-nanostructure embedded in a conductive polymer (PEDOT:PSS) [34]. Additionally, an array of aligned ferroelectric barium strontium titanate (BST) NWs acted as a wearable cooling device by using the electrocaloric effect of the NW array [42]. This array, encapsulated by PDMS, withstood a radius of curvature of 5 mm and 25% strain. NWs can also be incorporated into flexible FETs. By using sellotape as a substrate, an array of FETs based on CuPc organic NWs achieved a 3 mm bending radius [139].

#### 2.5.6. Energy Storage

Wearable electronics also include energy storage and harvesting devices. Such FHE could power and enable wireless communication. A common material for wearable energy storage applications is MnO_2_. One work developed a capacitive energy storage device by encapsulating molybdenum carbon nanofibers (Mo_2_C NFs) with MnO_2_ nanosheets via electrospinning [40]. Applied as a supercapacitor, the device achieved a specific capacitance of 430 F/g and 302 F/g at current densities of 0.1 A/g and 1 A/g, respectively, with 92.6% capacitance retention after 5000 charging cycles. Similarly, a supercapacitor used carbon nanofibers covered with MnO_2_ nanosheets and carbon nanofibers covered with graphene to build a wearable all-solid-state supercapacitor [116]. This supercapacitor indicated a specific capacitance up to 87.1 F/g and cycling voltammetry curves did not change despite different bending states. Other than fiber-shaped supercapacitors, MnO_2_-based flexible supercapacitors also can be designed in the shape of a sheet. Here, an MnO_2_ nanosheet grown on a nitrogen-doped graphene nanosheet with PVA-LiCl gel electrolyte created a supercapacitor capable of 305 F/g specific capacitance [41]. Another film-shaped supercapacitor involved the development of an amino-functionalized MWCNTs and MnO_2_ thin film composite as electrodes separated by a cellulose layer [140]. This design retained 90% of its capacitance after 2000 bending cycles at 90°. Another nanosheet was printed via nickel hydroxide (Ni(OH)_2_) ink coated on a carbon fiber yarn substrate in order to create a wearable energy storage device [141]. Graphite foam composites have also been applied to wearable supercapacitors which power sensors and other components. Multilevel porous graphite foams (MPGs) and MPG/Mn_3_O_4_ composites (MPGMs) were developed as a lightweight and flexible supercapacitor [142]. This supercapacitor indicated a capacitance of 53 F/g and exhibited a 90% and 80% capacitance retention after 10,000 charging cycles and 1000 bending cycles, respectively. In addition to capacitors, a recent study from the Wang group developed wearable energy harvesters that implemented a biofuel system using lactate in sweat [143]. This system, comprised of Au serpentine interconnects, joined CNT bioanodes and cathodes and maintained a power density of 1.2 mW/cm^2^ at 0.2 V with 50% strain. Soft elastomers also allow wearable arrays of rigid electrical components. An array of chip-scale batteries with serpentine PI/Cu/PI layered interconnects, when placed on an Ecoflex substrate with a Silbione interlayer, can withstand over 30% biaxial stretching [144]. In this work, a wireless temperature sensor was powered by the flexible system. A printable Ag-Zn battery using a temporary tattoo paper accommodated strain up to 11.1% [145]. Additionally, a rechargeable Zn-Ag_2_-O battery on polyurethane employed a hyper-elastic binder to achieve 100% strain while powering an attached LED [146].

### 2.6. Implantable FHE

**Figure 3:**
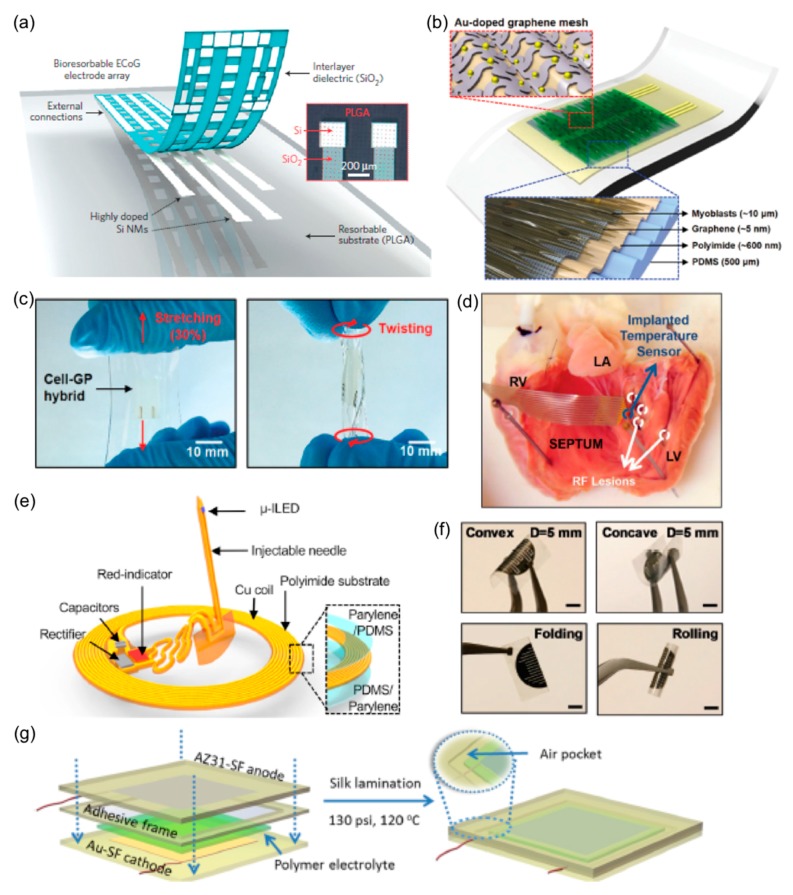
Implantable electronic systems. (**a**) Bioresorbable ECoG electrodes. Reprinted with permission from Macmillan Publishers Ltd.: Nature Materials [10]; (**b**) EMG electrode with Au-doped graphene mesh. Reprinted with permission from Reference [147], Copyright 2016, John Wiley and Sons; (**c**) Stretchability and flexibility of the serpentine-structured electrode in (**b**). Reprinted with permission from Reference [147], Copyright 2016, John Wiley and Sons; (**d**) Implantable cardiac sensor for monitoring temperature, thermal conductivity, and heat capacity. Reprinted from Reference [148], Copyright (2017), with permission from Elsevier; (**e**) Optogenetic device for wireless light delivery. Reprinted with permission from Reference [149], Copyright 2017, John Wiley and Sons; (**f**) Mechanically flexible, biodegradable microsupercapacitor. Reprinted with permission from Reference [49]. Copyright 2017, John Wiley and Sons; (**g**) Multi-layer illustration of a biodegradable battery with silk membrane. Reprinted with permission from Reference [150]. Copyright 2017 American Chemical Society.

Similar to wearable electronics, implantable FHE enabled by soft functional materials offer a number of applications in health monitoring, diagnostics, and therapeutics. Figure 3 shows a collection of representative examples of flexible-membrane based implantable electronics. Most of recent advancements in implantable systems are on biodegradable, transient materials; such resorbable electronics physically disappear at prescribed times and at controlled and guided rates. Table 4 collects a summarized list of implantable FHE, material characteristics, and functionality.

#### 2.6.1. Implantable Electrodes and Sensors

Bioresorbable ECoG electrodes have been developed with various materials. As shown in Figure 3a, phosphorus-doped Si nanomembranes (NMs) are used as an active material with a thin dielectric layer of SiO_2_ and a PLGA substrate [10]. This electrode conformed to a curvilinear surface for high-fidelity signal recording, while showing long-term stability. Porous graphene and Au wires on a PI substrate are also employed for ECoG electrodes [154]. This graphene is doped with nitric acid to improve its impedance. Another study presents an ECoG electrode array on a Cyclic Olefin Polymer (COP) substrate with gold electrodes [103]. Figure 3b shows a layout of a flexible EMG sensor [147]. The multi-layers of PI, Au-doped graphene, and aligned C2C12 myoblast sheet on the PDMS substrate offer a favorable in vivo interface with the skin. The mechanical properties of the electrode reaching 40% elongation and over 90° bending are demonstrated in Figure 3c. Another EMG sensor uses capacitively coupled silicon nanomembrane transistors with an ultrathin silicon dioxide layer as a dielectric layer [151]. Through polydopamine (PDA) reduction of GO, a hydrogel can be designed to be a self-healable, conductive, and biocompatible hydrogel with an extension ratio greater than 35 [155]. This hydrogel showed a variety of applications, including a finger motion sensor, self-adhesive electrode, intramuscular electrode, and cell stimulator.

An epicardial mesh for ECG recording and biventricular pacing was made of a functional composite of biocompatible styrene-butadiene-styrene rubber and ligand exchanged AgNWs [152]. With a mesh design, the device withstood the required radial strains of 90% to stimulate the ventricles and compared well for ECG recording. Graphene FETs to record potentials also have been developed on a PI membrane via a high throughput transfer technique [156]. By using an array of ZnO NWs coated with a gold film and encapsulated by PEDOT, impedance and signal-to-noise ratio significantly decreased due to increased surface area, which improved sensor performance [57]. To enhance the mechanical flexibility, the PI and Au-graphene hybrid were used as a substrate and an interconnection line, respectively. A study created injectable cardiac sensors for real-time monitoring of temperature and thermal transport properties with a flexible PET substrate, thin Au wires, and an epoxy encapsulation [148]. This flexible cardiac sensor is implanted in a heart as shown in Figure 3d. Another implantable sensor composed of a p(AM-co-PEGDA) core and a Ca alginate cladding to detect physical and optical changes in response to glucose. [157]. This fiber bent up to 80° with a 30% light intensity loss and indicated a readout rate of 1.33 mmol/L-min, which was 17 times greater than the required rate. One study designed an Mg-based biodegradable sacrificial layer for bonding with a flexible electrode to provide a necessary rigidity for implantation [158]. Once implanted, the physiological fluid dissolved the sacrificial layer, enabling conformal integration with the tissue.

#### 2.6.2. Actuators

Implantable actuators can be developed with soft materials. For example, a robotic sleeve to assist the heart used a low-modulus elastomer (Ecoflex) as an actuated material and hydrogel as an interlayer [76]. Additionally, a light delivering device for optogenetic purposes is shown in Figure 3e [149]. The implantable device consisting of a Cu coil and other miniaturized electronics is encapsulated by layers of parylene and PDMS, which is bendable up to 9 mm in radius and stretchable up to 51% elongation.

#### 2.6.3. Energy Storage and Circuit Components

Similar to wearable FHE, soft materials enable compliant and implantable energy storage devices. A biodegradable and flexible microsupercapacitor is demonstrated by implementing metal thin films, agarose hydrogel, and PLGA substrate (Figure 3f) [49]. The metal films that are fabricated with gold, tungsten, iron, and molybdenum shows an areal capacitance of 0.61 mF/cm^2^. Another study developed a supercapacitor to use in physiological fluids [159]. Here, aligned hydrophilic CNT sheets were applied to create CNT fibers as supercapacitor electrodes, while using the surrounding physiological fluid as an electrolyte. To develop the implantable, biodegradable battery in Figure 3g, silk fibroin films are utilized, including Mg-silk fibroin anode and Au-silk fibroin cathode [150]. The electrolyte consists of a silk fibroin-choline nitrate polymer and the device includes an encapsulated silk layer. Additionally, flexible and implantable energy harvesters have been developed; to implant an energy harvester into a heart, a PMN-PZT-Mn piezoelectric crystal is attached to a PET substrate via polyurethane [153]. This device works as a wireless ECG sensors that is bendable up to 2 cm in radius. Additionally, current research includes transient circuit components, such as the development of fully biodegradable logic circuits that utilize the biodegradability of silicon nanomembranes [160]. Using similar methods, this group demonstrated a multifunctional transient sensor [161]. A transient antenna with a tunable degradation utilized a PVA-TiO_2_ film substrate [162]. Other works involve studies of a PVA-based substrate where the dissolution rate was controlled by adding gelatin or sucrose [163].

## 3. Integration Strategies of Electronic Circuits for FHE

Continued engineering efforts in the study of soft functional materials and their system implementation allow ample opportunities to manufacture various FHE that are both mechanically and organically compatible with biological tissues. While these soft materials enable FHE to achieve multiple levels of mechanical compliance, they alone cannot realize fully functional and practical FHE. Core electronic components such as amplifiers, analog-to-digital converters, filters, microprocessors, memories, and multiplexers need to be embedded into a soft FHE for data acquisition, transmission, processing and active control. In this section, we review a few strategies to integrate essential electronic components onto flexible membrane circuits for mechanically compliant hybrid electronics.

### 3.1. Organic Electronics

**Figure 4:**
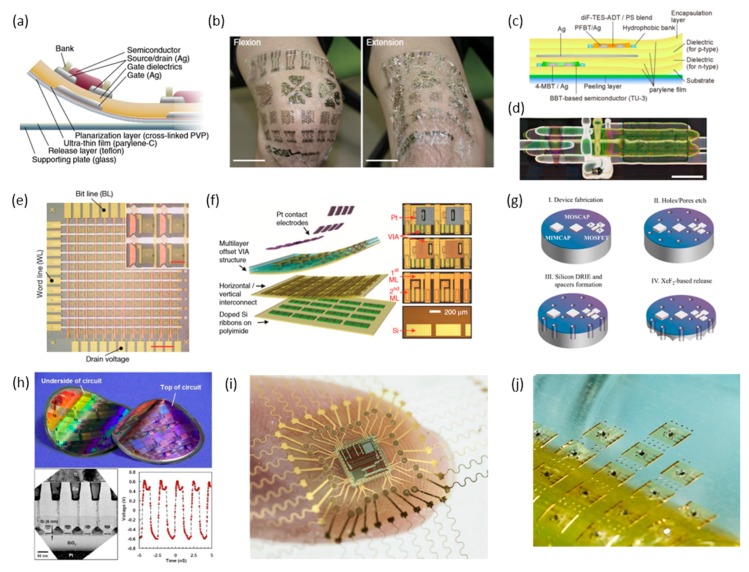
Integration strategies of electronic circuits for FHE. (**a**) Cross-sectional diagram of a fully-printed OTFT (organic thin-film transistors) device and (**b**) application of a thin organic film to a human knee. Scale bar, 4 cm. Reprinted with permission from Macmillan Publishers Ltd.: Nature Communications [164]; (**c**) Cross-sectional diagram and (**d**) photograph of an ultra-thin, fully-printed CMOS logic circuit. Scale bar, 500 µm. Reprint is in accordance with the Creative Commons Attribution 4.0 International License [165]; (**e**) Optical micrograph of a 12 × 12 tactile sensor array utilizing OTFTs as the switching transistors. Scale bar, 1 cm. The inset shows a magnified view of four pixels. Scale bar, 2 mm. Reprinted with permission from Macmillan Publishers Ltd.: Nature [166]; (**f**) Exploded view rendering of the flexible, high-density brain mapping device (left) and respective optical micrographs (right). Reprinted with permission from Macmillan Publishers Ltd.: Nature Communications [168]; (**g**) Process flow of XeF_2_-based Si exfoliation. Reprinted with permission from Reference [173]. Copyright (2014) American Chemical Society; (**h**) Flexible CMOS circuits formed by controlled spalling (top). Cross-section TEM image of the flexible circuit, stressor and handle layers (bottom left). Resulting voltage waveform of a 100 stage ring oscillator (bottom right). Reprint is in accordance with the Creative Commons Attribution 3.0 International License [170]; (**i**) Integration of a thinned die in a flexible substrate. Reprinted from Reference [171], Copyright (2015), with permission from Elsevier; (**j**) Scaled production of soft-adhesive electronics with surface mount chip components. Reprinted with permission from Reference [172], Copyright 2017, John Wiley and Sons.

Electronic devices consisting entirely of polymers have drawn significant interests due to their intrinsic mechanical flexibility. Since most organic materials can be processed in a solution form, a large-scale fabrication can be done by printing processes. In fully printed organic systems, the devices are formed by an additive manufacturing, thus no further subtractive steps are needed for structural patterning. For example, Tokito’s group has demonstrated the fabrication of fully-printed organic thin-film transistors (OTFT) and circuitry on ultra-thin parylene-C films (Figure 4a). They have showed a large-area printing (Figure 4b) on a single, continuous film, which allows the entire system to be worn over the target skin areas [164]. Complementary logic circuits (e.g., CMOS) employing both p-type and n-type OTFTs have been fabricated by a similar printing method (Figure 4c,d) [165]. Someya’s group also demonstrated the integration of OTFTs as switching transistors for an ultrathin, active-matrix tactile sensor, validating the potential of organic electronics for wearable devices (Figure 4e) [166]. Despite the manufacturing flexibility and scalability of organic electronics, a complete realization of modern electronic requirements solely by the use of organic materials has been challenging. First of all, acquiring electronic functions with fast switching speeds and high on-off ratio might be challenging due to the far less carrier mobilities of organic semiconductors than that of inorganic, single crystalline semiconductors. This limits the roles of organic electronics to low-speed tasks. Moreover, both structural and chemical stability of the organic materials needs to be further improved to achieve the device reliability, especially for body-implantable applications.

### 3.2. Inorganic Electronics

While typical semiconducting wafers are rigid and brittle, exfoliating them into thin layers with a thickness less than 25 µm yields mechanical bendability and flexibility [167]. Therefore, an implementation of thin, single crystalline semiconductor layers into flexible substrates would provide superb electronic properties, mostly owing to the carrier mobilities that are orders of magnitude higher than those of organic counterparts, allowing the circuits to be tasked with more demanding functions. Moreover, since modern photolithography technologies would be used for patterning, extremely dense array of transistors can be packed in a small area. Rogers’s group developed such materials processing method, enabling both exfoliation and manipulation of silicon nanomembranes based the use of a silicon-on-insulator (SOI) wafer [168]. As shown in Figure 4f, once high-thermal processes (oxidation and diffusion doping) are carried out on SOI wafers to define source and drain areas, the doped silicon layer can be released by removing the insulating oxide layer. The released layer is then transferred onto a flexible substrate to finalize device configuration through low-temperature processes including dielectric and metal depositions. Alternatively, as demonstrated by Hussain’s group, lower-cost silicon wafers with <100> crystal orientation can be processed with directional reactive ion etching, followed by isotropic etching with XeF2 to effectively release the top silicon layer (Figure 4g) [169]. Lastly, a process utilizing a controlled fracture across a wafer with use of a stressor layer can be used to peel away a layer of wafer containing completed CMOS circuits. The Bedell group showed that controlled spalling could successfully exfoliate 10 µm-thick top layer from a 300-mm silicon wafer by using a 6 µm-thick Ni stressor layer (Figure 4h) [170]. The spalling process utilizes the intrinsic materials properties, ensuring a complete removal of residual stresses, which can adversely affect circuit properties, requires extensive processing time and fabrication complexity.

### 3.3. Thinned Chips

Rather than exfoliating a thin layer of a wafer, diced individual silicon chips can be chemically and mechanically ground down to achieve required mechanical properties. The thinned chips can then be bonded to flexible substrates either with the active side facing down to the pre-patterned fan-out traces using conductive adhesives or with the active side facing upward followed by spin-on film deposition and establishing electrical connections via microfabrication. Jan Vanfleteren’s group demonstrated the integration of a 30 µm-thick, application-specific integrated circuit with a flexible substrate, enabling multi-channel brain recording and stimulation in small rats (Figure 4i) [171]. Similar to spalled electronic layers, the thinned chips can be sensitive to both internal and external stresses, and maturation of the technology needs to take place by further developing device validation processes.

### 3.4. Chip-Scale Packaging

Recent advancement in the electronic packaging technology has resulted in the production of chip packages with minimally added materials while establishing features, such as solder bumps, directly on the circuit side to enable chip integration. For example, ball grid array and chip scale package components can be considered as bare die chips with preformed solder balls either in non-array or array configurations, respectively. While this construction adds few tens of micrometers to the overall thickness, the solder-based approach provides the most robust electrical and mechanical connection between the chips and flexible substrates. Because these types of packages are commercially available, no additional processing steps are required for film integration. Moreover, multiple components, such as thick-film passive components and other solderable packages based on surface mount technology, can be assembled simultaneously by using an automatic pick-and-place tool. The manufacturing method introduced by Kim et al. [172] shows the potential of integrating commercial off-the-shelf chip components with a stretchable platform that is compatible with conventional soldering processes. The key features enabling this particular solderable and stretchable platform are (1) excellent solderability and compatibility with conventional surface mount technology, (2) ability of the assembled device to be directly integrated with a soft adhesive layer, and (3) scalability of the manufacturing method as described in Figure 4j.

## 4. Health Monitoring and Disease Diagnostics

**Figure 5:**
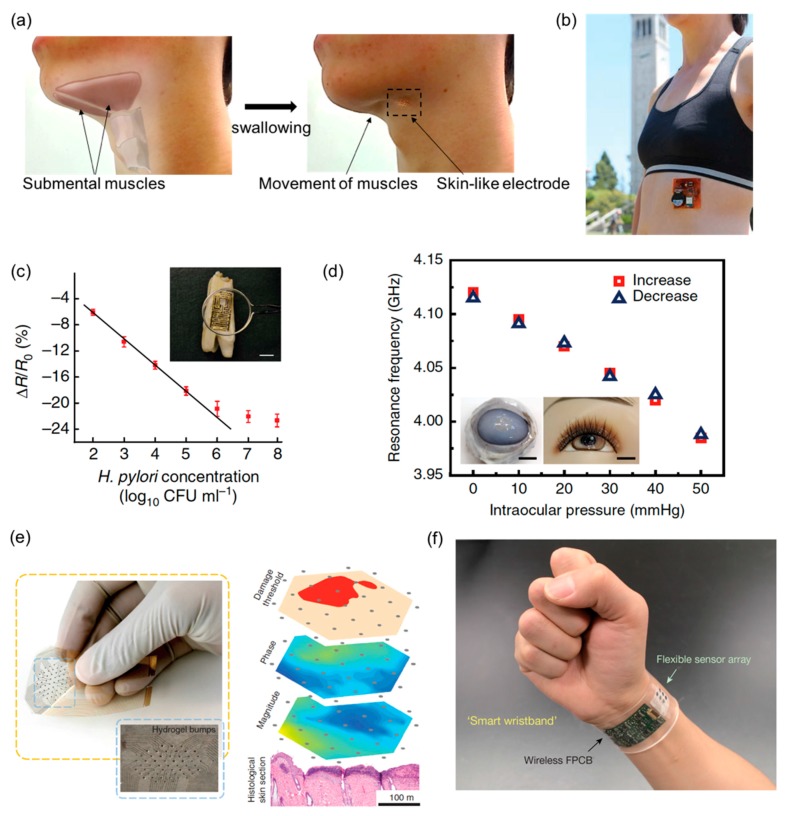
Health monitoring and disease diagnostic systems. (**a**) Illustration of targeted submental muscles on the chin and photos capturing the movement of the muscles upon swallowing activity. Reprinted with permission from [109], Copyright 2017, Scientific Reports; (**b**) Photographs of the wearable sensor patch mounted on a person’s lower left rib cage (left) and the component side of the patch (right). Reproduced from [174], Copyright 2016, John Wiley and Sons; (**c**) Percentage change in graphene resistance versus concentration of H. pylori cells with optical image of the graphene wireless sensor biotransferred onto the surface of a tooth (inset optical image). Reproduced from [175], Copyright 2012, Macmillan Publishers Ltd.: Nature Communications; (**d**) An inkjet printed array, showing the hexagonal configuration of 55 equally spaced gold electrodes; inset shows printed hydrogel bumps on the fabricated array (left). Schematic representation of the device operation for early detection of pressure ulcers (right). Reproduced from [176], Copyright 2015, Macmillan Publishers Ltd.: Nature Communications; (**e**) Frequency response of the sensor during a pressure cycle for ocular diagnostics. Inset shows photographs of the sensor transferred onto the contact lens worn by a bovine eyeball (left) and a mannequin eye (right). Scale bar, 1 cm. Reproduced from [29], Copyright 2017, Macmillan Publishers Ltd.: Nature Communications; (**f**) Photograph of a wearable flexible integrated sensing array on a subject’s writs, integrating the multiplexed sweat sensor array and the wireless flexible printed circuit board (left). Simultaneous system-level measurements (right). Reproduced from [177], Copyright 2016, Macmillan Publishers Ltd.: Nature.

Non-invasive, wearable FHE enable a portable, real-time, in vivo disease diagnosis and health monitoring due to intimate and conformal integration with the target sources such as the human skin, eyes, and mouth. Unlike conventional methods, wearable FHE can be directly mounted on various human body parts to continuously and closely monitor health conditions and disease related biomarkers in timely manner, without interrupting or limiting the user’s motions. This continuous physiological monitoring and intervention in a minimally invasive way would have direct benefit at early disease diagnosis and real-time monitoring of therapy, treatment, and health conditions. Figure 5 summarizes recent reports of FHE-enabled health monitoring and disease diagnosis applications.

Figure 5a shows a soft, highly compliant electrode for long-term wearable, high fidelity monitoring of difficulty in swallowing through EMG on the chin [109]. A swallow disorder is a common symptom of dysphagia. A skin-like electrode is fabricated by the combination of a wafer-scale microfabrication and material transfer printing. The resulting device mounted on the target location of the skin demonstrates clinical feasibility of the ergonomic electronics in rehabilitation for patients with dysphagia. Figure 5b captures a wearable sensor patch that brings together soft and hard electronics into a single platform using direct printing technique with gold and nickel oxide nanoparticle inks [174]. ECG electrodes and thermistor directly printed on a flexible polyimide substrate offer soft, low-modulus mechanics at the system level. The wearable sensor patch is mounted on a person’s lower left rib cage to measure ECG and skin temperature. Figure 5c presents graphene-based bacteria detection on tooth enamel. Graphene is printed onto water-soluble silk that allows intimate biotransfer onto a tooth [175]. A resonant coil is incorporated to eliminate the need for onboard power and external connections. The device demonstrates the remote monitoring of respiration and bacteria detection in saliva with detection limit down to a single bacterium. Figure 5d integrates transparent, stretchable, and multifunctional sensors onto wearable soft contact lenses for the wireless detection of glucose and intraocular pressure with high-sensitivity for ocular diagnostics [29]. A multifunctional contact lens sensor is designed to monitor glucose within tears, as well as intraocular pressure using the resistance and capacitance of the electronic device. The hybrid of graphene and metal nanowires offers sufficient transparency (>91%) and stretchability (~25%) that ensure reliable, comfort, and unobstructed vision when users have the soft contact lens on. Its reliable operation is demonstrated both in vivo and in vitro studies using a live rabbit and bovine eyeball.

A flexible, electronic device shown in Figure 5e offers non-invasive mapping of pressure-induced tissue damage [176]. The flexible electrode array is placed on a wound and the electrical impedance is collected for each pair of neighboring electrodes. A map of impedance magnitude, phase angle and damage threshold (indicated in red in Figure 5e) are constructed based on the location of each measurement pair. The results show the feasibility of an automated, non-invasive ‘smart-bandage’ for early detection of pressure ulcers. Figure 5f shows a fully integrated FHE composed of sensor array for simultaneous and selective multiplexed analysis in sweat including glucose, lactate, electrolytes, and skin temperature [177]. The platform enables a wide range of personalized diagnostic and physiological monitoring applications. Wearable FHE sensors have the potential to play a major role in the wireless, continuous, and noninvasive monitoring of physiological conditions, as well as the detection of biomarkers associated with diseases. In particular, multiplexing sensing elements show substantial promise for next-generation medical devices to provide a tangible impact on health and wellness.

## 5. Human-Machine Interfaces (HMI)

**Figure 6:**
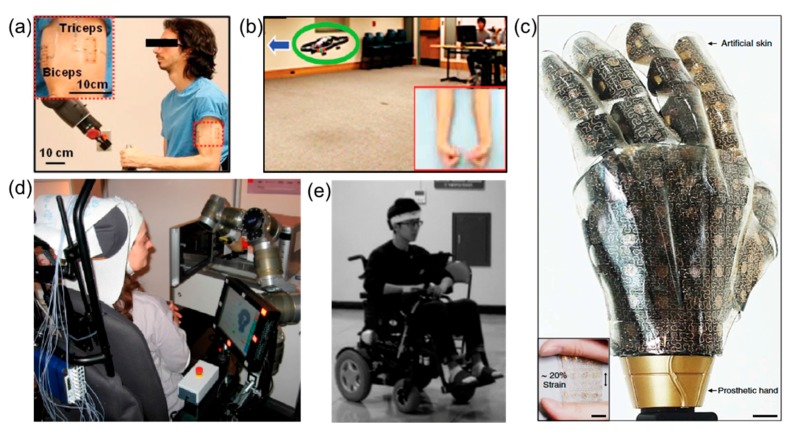
Flexible hybrid electronics for applications in human-machine interfaces. (**a**) EMG-enabled control of a humanoid robot. Reprinted with permission from Reference [178], Copyright 2016, John Wiley and Sons; (**b**) Bimanual gestures and their EMG signals, interfacing with a quadcopter. Reprinted with permission from Reference [7], Copyright 2013, John Wiley and Sons; (**c**) Sensor-laden bionic hand, instrumented with silicon nanoribbon. Reprinted with permission from Macmillan Publishers Ltd.: Nature Communications [179]; (**d**) Wearable headset and EEG (electroencephalogram) recording for a brain-interfaced system. Reprinted with permission from Reference [180]. Copyright 2015, MDPI; (**e**) Recording of EOG via a wearable forehead system for a wheelchair control. Reprinted with permission from Reference [181]. Copyright 2017, MDPI.

Recent remarkable progress in the development of wearable FHE has offered non-invasive, highly sensitive interactions between human and machines. Mechanically compliant FHE enable conformal contact to the human skin in a non-invasive way, while recording important physiological data such as EMG, EOG, and EEG. The biopotentials, measured from the skin-mounted electrodes, can be used to control a humanoid robot, drone, prosthetic hand, display interface, electronic wheelchair, and more. Figure 6 captures representative examples of recent HMI applications, controlled by FHE-enabled biopotentials. The main advantage of FHE in such examples is their portable, comfortable, and ergonomic arrangements via a low-profile, miniaturized electronic circuit, conformal electrodes, and data recording and management system.

A soft, skin-like sensor system in Figure 6a [178], directly mounted on the skin via van der Waals interactions, offers unobtrusive, comfortable HMI for sensorimotor prosthetic control of a humanoid robot. The multifunctional device has open-mesh structured, microfabricated electrode and sensor, integrated on an elastomeric membrane, which is mechanically flexible and stretchable to accommodate the strain from the skin deformation. The gold nanomembrane-based system has advantages of simultaneous sensing and electrical stimulation, which allows sensorimotor control of a robot arm. With the stimulation feedback, a subject who wears the device can grip a water bottle in a controlled manner to prevent collapse. A similar device [7] that makes an intimate contact to the skin (Figure 6b) offers a high-fidelity recording of surface EMG on forearms. Bimanual gestures, recorded by two sets of electrodes on forearms generate different signal commands to control a drone (e.g., quadcopter). There were four commands, generated by a signal classification algorithm that used EMG data on the skin. With an optimized electrode design and placement on the target muscle, this work demonstrated 91.1% classification accuracy with four classes.

Figure 6c presents a “smart” prosthetic hand [179] with artificial skin and embedded soft sensors, enabled by stretchable silicon nanoribbon on a silicone elastomer (PDMS). A high performance, single crystalline silicon designed a highly sensitive device with strain, pressure, and temperature sensors. The electronic skin on the prosthetic hand with the soft sensor package demonstrated capabilities of hand shaking, keyboard typing, ball grasping, and feeling surface temperature in daily lives. Another wearable set of FHE embedded in a headset [180] with EEG electrodes demonstrated the feasibility of hybrid brain-controlled computer (Figure 6d). In this study, the wearable head set recorded steady-state visually evoked potentials to control a computer interface, which can be directly usable for severely disabled people who cannot use their arms and hands. The event-related synchronization of EEG can be used in many applications such as text spelling interface, computer control, wheelchair control, and more. Among those, hands-free control of an electronic wheelchair has gained a great interest in rehabilitation and aging societies. Patients with Parkinson’s disease or amyotrophic lateral sclerosis experience paralyzed voluntary muscles and significantly reduced motor strength. In addition to the EEG-base method, eye movement can be utilized to control a wheelchair. A wearable FHE shown in Figure 6e [181] enables a non-invasive, comfortable arrangements to measure eye movements via EOG recording. In this work, the wearable system included forehead EOG electrodes on a headband along with Bluetooth-based wireless telemetry. A subject wearing the electrode system successfully drove a power wheelchair through an 8-shaped driving course without collision with obstacles. Collectively, soft, lightweight materials play a key role to design wearable, comfortable FHE. The mechanical compliance of the soft sensors makes conformal and intimate contact to the skin for high-fidelity recording of non-invasive biopotentials for various HMI applications.

## 6. Conclusions and Outlook

Recent progress in soft functional materials has enabled advances in wearable and implantable electronics. In this review, soft materials and designs for sensing and substrate components, along with the facilitated electronics and applications, have been summarized. Silicone elastomers and other soft, organic substrate materials, such as silk fibroin, have resulted in increasingly flexible and stretchable electronics, improving device functionality and increasing the breadth of potential applications. Additionally, a number of these materials have indicated necessary biocompatibility and biodegradability, allowing for applications in transient electronic needs. Likewise, sensing materials, such as CNTs, graphene, hydrogels, and nanostructures, have improved the mechanical and electrical characteristics of FHE. Sensing materials generally limit the mechanical compliance, but integration with soft substrates and recent advances have improved mechanical properties while maintaining favorable electrical properties. These advancements in soft material-enabled wearable and implantable electronics have improved functionality and allowed applications in different areas, including healthcare, disease diagnostics, and human-machine interfaces.

To continue progress in the advancement of wearable and implantable FHE, a variety of challenges and opportunities is still being addressed. Further development of soft material-based sensing components is required to offer smaller form factors of functional devices. New addition of self-healing materials will expand the current mechanical and application limits of FHE. For hard-soft device configurations that combine both compliant elastomers and rigid electronic components, advanced manufacturing methods are needed to provide robust mechanical and electrical hybridization of two types of materials. Improved technologies of multi-scale integration of hybrid materials will reduce the current gaps in performance variation between materials. While currently limited, further development of miniaturized and more efficient wireless powering and communication technologies will allow continuous and long-term usage of wearable and implantable systems. Additionally, continued investigation of biodegradable materials will broaden the applicability of transient electronics for implantable systems. Direct coupling of soft devices with cells and tissues will improve in vivo interfaces. Moving forward, continued development and integration of soft, functional sensing and substrate materials will heighten the functionality and applicability of FHE.

## Figures and Tables

**Table 1:** Representative sensing materials for wearable and implantable FHE (flexible hybrid electronics).

Sensing Material	Type	Biocompatible/Biodegradable ^1^
Carbon Nanotube	Organic	Yes/Yes ^2^ [44]
Graphene	Inorganic	Yes/No ^3^ [45,46,47]
Hydrogel	Organic/Inorganic	Yes/Yes ^4^ [48,49,50]
Liquid Metal (EGaIn)	Inorganic	Yes/No ^5^ [51]
Nanosheet and Thin Film (MnO_2_, Mn, Mg, Si)	Inorganic	Yes/Yes ^6^ [52,53,54,55]
Nanowire (Ag, ZnO, Si, Au, BaTiO_3_, Ni)	Inorganic	Yes/No ^7^ [56,57,58,59,60,61]
Conducting Polymer (PEDOT:PSS)	Organic	Yes/No ^8^ [62]

^1^ Biocompatibility is defined as the ability to biologically interact without adverse reactions, inflammatory responses, and toxicity. Biodegradability is considered being degradable, via different methods, within the human body without toxic effects [44]; ^2^ Surface and chemical functionalization, and designing structural defects in CNTs can be applied to create biocompatible and biodegradable CNTs [44]; ^3^ Biocompatibility studies of graphene composites have indicated bacterial and cell viability decrease slightly after exposure and in vivo studies, although limited, show resulting inflammation [45]. A second review suggests similar results, but states biocompatibility can be improved with surface functionalization [46]. Graphene surfaces have been shown to be biocompatible with neuronal cells and do not impact neuronal signal properties [47]; ^4^ Hydrogels are typically biocompatible and biodegradable, and can be tailored for applications [48,49,50]; ^5^ The common liquid metal of EGaIn is biocompatible [51]; ^6^ MnO_2_: MnO_2_ has shown low cytotoxicity, as Mn is an essential element in the body, and nanosheets degraded in the presence of glutathione [52,53]. Mg: Mg alloys are established biocompatible materials and biodegrade via corrosion [54]. Si: Silicon nanomembranes indicated no cytotoxicity during controlled dissolution in aqueous solutions [55]; ^7^ Ag: AgNWs exhibited no cytotoxicity with lung adenocarcinoma cells and lung normal fibroblasts [56]. ZnO: Biocompatibility was tested with a calcein and propidium iodide assay and less than 4% of cells died [57]. BaTiO3: Foam structure exhibited no short term cytotoxicity to mouse osteoblasts and did not promote a significant inflammatory response [58]. Si: Porous silicon NWs degraded in PBS [59]. Au: For different surface modifications and aspect ratios, Au NW toxicity was tested on fibroblasts and HeLa cells, and indicated potential for cell viability and low toxicity dependent on design [60]. Ni: Nickel NWs showed minimal toxicity to THP-1 cell macrophages over short timespans [61]; ^8^ Cell viability with PEDOT:PSS was determined for a variety of cell types [62].

**Table 2:** Substrate materials and their properties for wearable and implantable FHE.

Substrate Material	Organic/Inorganic	Young’s Modulus/% Elongation at Break	Biocompatible/Biodegradable
Silicone elastomer (Ecoflex 00-30)	Organic	0.07 MPa/900% [77]	Y/N ^1^ [78]
Silicone elastomer (Sylgard 184)	Organic	1.32–2.97 MPa [79]/120% [80]	Y/N ^2^ [81]
Silicone elastomer (Silbione LSR 4330)	Organic	1.38 MPa/750% [82]	Y/N ^3^ [83]
Parylene (VSI Parylene C)	Organic	2800 MPa/200% [84]	Y/N ^4^ [85,86]
Polyethylene terephthalate (PET)	Organic	230 MPa/120% [87]	Y/N ^5^ [88,89,90]
Polycaprolactone (PCL)	Organic	340.2 MPa/853.8% [91]	Y/Y ^6^ [92,93]
Polyimide (PI)	Organic	280 MPa/80% [87]	Y/N ^7^ [94]
Polyethylene naphthalate (PEN)	Organic	280 MPa/90% [87]	Y/N ^8^ [95]
Polyethersulfone (PES)	Organic	2654.5 MPa/100% [96]	Y/N ^9^ [97]
Polytetrafluoroethylene (PTFE)	Organic	0.06 MPa/400% [98]	Y/N ^10^ [99]
Poly(lactic-co-glycolic acid) (PLGA)	Organic	2000 MPa/3–10% [100]	Y/Y ^11^ [101]
Cyclic olefin polymer (Zeonor 1020R)	Organic	2100 MPa/90% [102]	Y/N ^12^ [103]
Silk fibroin	Organic	2500 MPa/2.1% (dry) 16.7 MPa/127.8% (wet) [104]	Y/Y ^13^ [105,106]

^1^ Ecoflex was biocompatible with neuronal-like cells, indicating no cell death or cytotoxic effects [78]; ^2^ Biocompatibility of a Sylgard coating was tested with fibroblast cells and in vivo, and exhibited high cell viability and minimal inflammation [81]; ^3^ Biocompatibility indicated on material data sheet [83]; ^4^ Parylene C has proven biocompatibility, including intraocular, and can be improved with specific treatments and processes [85,86]; ^5^ One study found electrospun PET meshes caused large foreign body reaction, but indicated that improving the nanofiber dimensions and surface-to-volume ratio may diminish the reaction [88]. Treating the surface of PET prevented toxic effects on human endothelial cells and fibroblast cells [89,90]; ^6^ PCL indicated biocompatibility, including with mouse fibroblasts, and is biodegradable via hydrolytic degradation [92,93]; ^7^ PI exhibited similar biocompatibility as PCL when it was implanted in rat sciatic nerve and resulted in inflammation up to 4–8 weeks before tissue recovered [94]; ^8^ PEN biocompatibility was tested with culturing of osteoblast-like cells [95]; ^9^ Cytocompatibility of PES membranes with human liver cells was confirmed via cell viability and adhesion [97]; ^10^ Biocompatibility, including low cytotoxicity and cell activation, of PTFE was confirmed with endothelial cells and macrophages [99]; ^11^ PLGA indicated no cytotoxicity, although high concentrations of degradation products, due to increased degradation rates, resulted in toxic effects [101]; ^12^ COP is considered to be biocompatible, although studies are lacking [103]; ^13^ Silk fibroin indicates no cytotoxicity and has adjustable degradation rates by altering crystallinity and molecular weights [105,106].

**Table 3:** Summary of wearable FHE, material characteristics, and functionality.

Device Type	Sensing Material	Application	Substrate Material	Target Signal	Sensitivity	Flexibility	Stretchability	Reference (Year)
Strain Sensor	MWCNT	Motion, Bending	Ecoflex	Resistance	1.5 GF	-	300%	[112] (2017)
	EGaIn Liquid	Motion, Contact	Ecoflex Microtubules	Resistance	-	-	750%	[107] (2017)
	CS-PDMS	Blood Pulse, Breathing,	PDMS	Resistance, Temperature	GF 1.78	180°	228%	[74] (2016)
	Graphite Flake Sheath and Silk Fiber Core	Joint Motion, Multiaxial	Ecoflex	Resistance	14.5 GF	-	15%	[66] (2016)
	Self-healing SWCNT-Hydrogel	Bending	VHB Mounting Tape	Resistance	GF 0.24 (100% Strain), GF 1.51 (1000% Strain)	540° Twisting, 150° Bending	1000%	[69] (2017)
Pressure Sensor	GPN	Blood Pressure	PDMS	Resistance	0.09/kPa	-	40%	[37] (2016)
Light Sensor	Ionic Liquid, PU fiber, SWCNT, Au film	Electronic Skin	Ecoflex	Conductivity	2.4 mW	90°	50%	[63] (2017)
Temperature	PEIE/CNT-PDMS. Ag electrode	Healthcare Patch	PET	Resistance, Voltage	0.85%/°C,	-	-	[113] (2017)
Sweat Sensor	InGaZnO ISFET, PI, CNT/PEDOT:PSS	Healthcare and Sports	PET	Current, Resistance	51.2 mV/pH	10 mm Radius	-	[114] (2017)
	Hydrogel, Ag/AgCl Electrode	Fitness Monitoring	PET	Voltage	52.8 mV/decade	-	-	[70] (2017)
Electrode	Au	EOG, Eye Movement	PI	Voltage	13.3 µV/°	0.5 mm Radius	30%	[8] (2017)
Antenna	Ag-PDMS	Wireless Communication		Conductivity	-	-	20%	[115] (2017)
QD Display	QDs	Sensor Display, Touch Sensor	Parylene	Intensity	-	180°	-	[111] (2017)
Cooling Device	BaSrTiO Nanowires	Cooling	PDMS	-	-	5 mm Radius	25%	[42] (2016)
Supercapacitor	MnO_2_ Nanosheet, Carbon Fiber, Graphene, PVA	Energy Storage	Cotton Textile					[116] (2016)

**Table 4:** Summary of implantable FHE, material characteristics, and functionality.

Device Type	Sensing Material	Application	Substrate Material	Target Signal	Sensitivity	Flexibility	Stretchability	Reference (Year)
Electrode	Myoblasts, Au-Graphene	EMG, Stimulation, Therapy	PI, PDMS	Voltage	-	-	40%	[147] (2016)
	Si nanomembranes	Electrophysiological Mapping	PI	Voltage, Current	-	5 mm radius	-	[151] (2017)
	Doped Si Nanomembranes	Monitor Brain, Muscle, Organ Activity	PLGA	Voltage	-	1 mm radius	-	[10] (2016)
	LE-AgNW	ECG, Biventricular Pacing	SBS Rubber	Voltage, Contractility	-	-	-	[152] (2016)
Cardiac Temperature Sensor	Au	Lesion Characterization	PET	-	0.26%/°C	21 N/m Bending Stiffness	-	[148] (2016)
Optogenetic Light Delivery	Cu	Optogenetics	PI, Parylene, PDMS	Output Power	-	6 mm radius	-	[149] (2017)
Biodegradable Microsupercapacitor	W, Fe, Mo, NaCl-Hydrogel	Power Storage	PLGA	Capacitance	-	5 mm diameter	-	[49] (2017)
Biodegradable Battery	Mg	Power Supply	Silk Fibroin	-	0.06 mAh/cm^2^ (Specific Capacity)	-	98%	[150] (2017)
Energy Harvester	PMN-PZT-Mn	ECG, Wireless Data Transmission	PET, PU	Voltage	-	9.95^−5^ N/m Bending Stiffness	-	[153] (2017)
